# Probabilistic Risk Assessment of Polioencephalomalacia from Texas Beef Consuming Rations Containing Multiple Sources of Dietary Sulfur

**DOI:** 10.3390/ani14162400

**Published:** 2024-08-19

**Authors:** Ashli A. Brown, Timothy Herrman

**Affiliations:** 1 Office of the Texas State Chemist, Texas A&M AgriLife Research, Texas A&M University System, College Station, TX 77843, USA; 2 Department of Soil and Crop Sciences, Interdisciplinary Faculty of Toxicology, Texas A&M University, College Station, TX 77843, USA; tjh@otsc.tamu.edu

**Keywords:** beef cattle, effective neutral detergent fiber, hydrogen sulfide, polioencephalomalacia, sulfur

## Abstract

**Simple Summary:**

In Texas beef markets, the expansion of ethanol production has increased the demand for and utilization of biofuel co-products, like dried distiller grains with solubles, introducing highly variable concentrations of nutrients, such as sulfur, known to impact cattle health. The risk of sulfur toxicity for Texas cattle was estimated using two mathematical nutrition models at two production stages across twelve geographic districts. The assessment identified cattle raised in the South Plains region of northwest Texas as the most susceptible to sulfur toxicity, with those in the finishing production stage being more sensitive. Results also highlighted feed ingredients and water wells by district that significantly contributed to the risk of sulfur toxicity. This research further helps better manage the risk of sulfur toxicity by providing tools translatable nationwide that properly balance beef diets and calculate anticipated sulfur exposure.

**Abstract:**

The purpose of this probabilistic assessment was to estimate the risk of sulfur-induced polioencephalomalacia (S-PEM) for beef raised across Texas, from a dietary perspective. Ruminant nutritionists in Amarillo, TX, formulated two typical nutritional regimens based on cattle production stages, each containing six feed ingredients and well water. The Office of the Texas State Chemist (OTSC), National Research Council (NRC), and the published literature provided S data for feed ingredients. The Texas Water Development Board provided data for S content in Texas well water, categorized into twelve districts established by the Texas A&M AgriLife Research Extension Service. The S-PEM risk was estimated at five different eNDF levels ranging from 0% to 8% in 2% increments, using rumen degradable S (RDS) as an input value. Findings identified cattle raised in the South Plains district as the most susceptible population to S toxicity, with beef in the finishing production stage experiencing increased sensitivity. The most potential (MP) risk scenario suggested that the S-PEM risk could reach 28.5% for growers and 100% for finishers. Results further revealed that when S concentrations in well water exceeded 14.5 mg/L, water became the greatest contributor to RDS content for Texas beef, suggesting that high S content in well water is the most prominent concern for Texas beef.

## 1. Introduction

Texas is a top cattle producer in the United States, boasting the heaviest concentration of feedlot operations and the most extensive range of farmlands that cover over 127 million acres [1]. Cattle production has held significant cultural, historical, and economic importance in the Lone Star state for decades, leading agricultural sales by 50% and generating more than $12.3 billion annually [1]. Favorable climate conditions, diverse geography, abundant resource availability, and vast pasturelands make Texas a cornerstone of U.S. beef production, representing 14% of the nation’s beef cows [1,2]. Recent expansion in ethanol production has significantly increased the utilization of biofuel co-products, like dried distiller grains with solubles, as nutrient-dense, economical alternatives for corn and soybean-based feed ingredients in animal feed markets [2,3]. However, DDGS are not homogenous and often contain highly variable sulfur content, limiting their inclusion in ruminant diets [2,3].

Sulfur (S) is an essential macronutrient, comprising 0.15% of an animal’s body weight and serving several biological functions vital to life [4,5]. For beef cattle, acceptable levels of S maintain stable growth and normal ruminal bacteria reproduction, supporting feed digestion [4,5]. Excess sulfur results in the ruminal accumulation of endogenous hydrogen sulfide (H_2_S) that is eructed, inhaled, and absorbed into the pulmonary blood system, with some concentrations reaching the brain before hepatic detoxification. In the brain, high H_2_S concentrations inhibit the last enzyme, cytochrome c oxidase, of the respiratory electron transport chain, resulting in reduced feed intake, delayed growth rate, decreased trace mineral absorption, and sulfur-induced polioencephalomalacia [3,4,5,6].

Sulfur-induced polioencephalomalacia (S-PEM) is a neurological condition characterized pathologically by cerebral cortex necrosis and clinically by recumbency, head pressing, nystagmus, blindness, and seizure activity in ruminants [7,8]. Treatment for S-PEM is symptomatic and involves thiamine administration; however, full recovery is unlikely, and cattle may still display clinical S-PEM indications. The USDA Federal Meat Inspection Act (FIMA) and the Texas Administrative Code §221.14 prohibit sick or unsound animals exhibiting abnormal conditions from custom and commercial slaughter for human food consumption, and thus, excess dietary S has the potential to negatively impact the Texas beef economy [9,10].

Although a magic potion to reverse the detrimental health effects of S toxicity does not exist, ruminant nutritionists can prevent S-PEM by understanding the ruminal availability of dietary S and ensuring sufficient roughage is incorporated into diets [3]. The probability of S-PEM being reduced with increased neutral detergent fiber (NDF) in diets, the carbohydrate portion of feedstuffs, was demonstrated in [11]. Scientists also identified rumen degradable sulfur (RDS) as a better measure for S-PEM than total dietary S (TDS) due to consideration of inorganic S components directly contributing to ruminal H_2_S accumulation [11]. It is known that many parts of Texas experience elevated S levels in water; however, the study by Nichols et al. (2013), similar to most risk assessments, did not consider water, the most significant exposure source recognized by the National Research Council (NRC). Furthermore, the NRC 2000 Feed Library reveals that all roughages do not contain fiber that successfully supports rumen motility and feed digestion, suggesting that effective NDF (eNDF), the percentage of NDF that sufficiently stimulates microbial protein activity, is a more precise measure of fiber in diets. Thus, there exists a paucity of research utilizing S data from feed and water and scientific advances concerning rumen motility to assess the S-PEM risk for nation’s largest cattle production region.

The purpose of this work was to estimate the potential risk of S-PEM from Texas beef consuming diets containing multiple sources of S at five different forage fiber levels. These proposed efforts further intend to shed light on the feed ingredients and Texas water wells that exacerbate S-PEM risk in hope of reducing excess S exposure and protecting the economic viability of Texas beef cattle.

## 2. Materials and Methods

### 2.1. Formulated Diets and Water Requirements

Ruminant nutritionists from Amarillo, TX, USA (Dr. Travis Whitney and Pake Ebert) formulated two DDGS-based diets that captured the transition of Texas beef from high-fiber, limited-concentrate grower feeds that improve productivity and increase dry matter intake to low-fiber, high-energy finisher feeds that rapidly fatten livestock to reach optimal beef quality grades before custom or commercial slaughter. The typical diet for growers contained 18.2% DDGS, 36.1% dry-rolled corn (DRC), 13.5% sorghum silage, 27.1% cotton hulls, 2.2% vitamin–mineral (VTM) supplement, and 2.9% liquid feed blend (LFB). The regular diet for finishers comprised 16.8% DDGS, 68.2% steam-flaked corn (SFC), 3.3% sorghum silage, 6.7% cotton hulls, 1.8% VTM supplement, and 3.2% fat. These formulated diets were not directly dependent on livestock BW. Instead, ranges of BW served as indicators of beef life stage and production type, which determined the appropriate diet composition and water requirements. As supported by NRC 2000, the BW for growers ranged from 400 to 600 lbs., not exceeding 800 lbs., while finishers weighed 600 to 800 lbs., not exceeding 1000 lbs [4].

Aside from dietary regimens, beef cattle require water to regulate body temperature, mineral homeostasis, macronutrient hydrolysis, digestion, metabolism, and waste excretion [4]. The NRC 2000 recommends the approximate water needs of beef cattle based on life stage, production type, and environmental temperature. The daily water requirements for growers range from 19.4 to 47 L, with a 29 L average [4]. Finishers require slightly more water, ranging from 27.7 to 66 L at an average of 40.8 L/d [4].

### 2.2. Sources of S

Feed ingredients and water wells were the examined S sources. The Office of the Texas State Chemist (OTSC) located in College Station, TX, USA provided S data for 331 DDGS samples collected in Texas during the 2012 to 2019 sampling years. Qualified OTSC personnel analyzed these samples utilizing in-house elemental analysis on an Agilent 5110 Inductively Coupled Plasma-Optical Emission Spectrometer (ICP-OES), sourced from Santa Clara, CA, USA [12]. The sample analysis involved nitric acid (HNO_3_) digestion, deionized water clean-up, and 3% HNO_3_ dilution. Emission lines for measuring S were specified at roughly 181.9 nm.

Ruminant nutritionists supplied the S content of LFB, while the NRC 2000 Feed Library provided S concentrations for all remaining feed ingredients of the DDGS-based diets [4].

The Texas Water Development Board (TWDB) Groundwater Database (GWDB) supplied sulfate (SO_4_^2^) levels of nearly 140,000 Texas water wells by county. These concentrations were averaged, converted to S, and aggregated into twelve districts outlined by the Texas A&M AgriLife Research Extension Service. The districts were as follows: D1—Panhandle, D2—South Plains, D3—Rolling Plains, D4—North, D5—East, D6—Far West, D7—West Central, D8—Central, D9—Southeast, D10—Southwest, D11—Coastal Blend, and D12—South. Each district was composed of at least 20 counties. The total number of samples varied by district but typically included over 5500 samples.

### 2.3. Nutrient Composition

Table 1 presents the nutritional data for feedstuffs incorporated in grower and finisher diets, sourced from the NRC 2000 Feed Library headquartered in Washington, DC, USA. Crude protein (CP) represents the protein content of ingredients, while the rumen degradable protein (RDP) refers to the portion of protein available for rumen degradation by microbial enzymes. Sulfur-containing amino acids refer to the total number of organic compounds in feedstuffs containing S.

The NRC 2005 identified methionine (Met) and cysteine (Cys) as the most common SAAs in preselected feedstuffs, compromising 21.5% and 26.5% S, respectively [5]. However, the NRC 2000 feed library lacked data on Cys for each feed ingredient. Thus, Met was the only SAA considered in this study. Neutral detergent fiber (NDF) is the carbohydrate portion of feedstuffs, representing fiber content insoluble in neutral detergent. In contrast, effective neutral detergent fiber (eNDF) refers to the percentage of NDF that sufficiently stimulates rumination, salivation, and microbial protein activity.

### 2.4. Mathematical Nutrition Models

The Palisade @RISK decision analysis software (Version 8.0) was used to construct the mathematical nutrition models, which combined formulated beef diets, water requirements, S content, and other nutritional data to estimate the potential dietary RDS exposure for growers and finishers raised across Texas. Table 2 provides the key formulas for calculating the amount of S ruminally available while accounting for all inorganic and organic sources of S. Each formula was dependent on values calculated in prior formulas. The first computation estimated the amount of rumen-undegradable protein (RUP) for each feed ingredient by subtracting the degradable portion, RDP, from 100. The RUP content was then multiplied by the percentage of Met and S in Met to determine the organic portion of S, SAA, for each feed ingredient. Multiplying the average S content in water wells per district by cattle water intake rates and a 1 × 10^−4^ conversion factor provided the total S exposure from Texas water wells in percentage form. Total dietary S (TDS) for grower and finisher diets was calculated by adding the average TS of each district to the sum of inorganic and organic S content of each feed ingredient.

Computing RUP, SAA, TS, and TDS provided all the values required for estimating the RDS content of Texas beef diets utilizing a formula adapted from [11] to subtract the product of multiplying RUP and SAA from TDS to derive RDS. The RDS formula was provided by [13], assuming all sources of inorganic S are subject to ruminal reduction to S^2−^, which produces endogenous H_2_S as the ruminal pH decreases. Since the ruminal degradation affects the profile of amino acids, this formula also assumes organic S sources, such as SAA, are 100% available for ruminal fermentation [13]. This concept did not account for S sources embodied in bacterial mass, assuming they were 100% unavailable for SRB reduction to S^2−^ since bacterial CP escapes the reticulorumen [13]. Other assumptions involved 100% availability for metabolic intermediates of S-containing amino acids and lipids with unknown degradation characteristics. The RDS computation was not used for DDGS and water wells, which included only ruminally available inorganic S, causing RDS to equal TDS.

Suppose finishers housed in the Panhandle (D1) consume mixed feeds composed of 71.4% SFC that contains 0.14% inorganic S, 9.8% CP, 43% RDP, 1.12% Met (% RUP), and 21.5% S in Met, then the potential RDS exposure from SFC and well water can be calculated as follows:

RUP = 100% − [(43% RDP) × (9.8% CP)] = 95.8% RUP;

SAA = (71.4% SFC) × (1.12% Met) × (95.8% RUP) × (21.5% S in Met) = 0.16% SAA;

TS exposure from water wells = 67.6 (mg/L) × 66 L × 1/10,000 = 0.4% TS in well water;

TDS = [(71.4% SFC) × (0.16% SAA + 0.14% inorganic S)] + (0.4% TS in well water) = 0.66% TDS;

RDS = 0.66% TDS − (95.8% UIP × 0.16% SAA) = 0.5% RDS.

### 2.5. Risk Characterization

To characterize the S-PEM risk, RDS concentrations estimated in the mathematical nutrition models were inserted into exponential equations that were adopted from [11] revealing the probability of S-PEM at five different eNDF levels ranging from 0% to 8% in 2% increments, with 4% eNDF being “normal”. 

The S-PEM risk for Texas growers and finishers was calculated based on two unique exposure scenarios: most potential (MP) and most significant (MS) risks. Table 3 displays the feed composition for grower and finisher DDGS-based diets in each risk scenario.

The MP scenario considered diets formulated by a ruminant nutritionist, with S content averaged for each feed ingredient and S content in Texas water well averaged for the twelve districts to represent the most likely risk. Since expert opinion revealed that the maximum DDGS inclusion rate for feedlots was 30%, a fixed DDGS inclusion rate of 30% and maximum concentrations of each feed ingredient were considered in the MS scenario, reflecting the worst-case scenario.

### 2.6. Sensitivity Analysis

Two sensitivity analyses were conducted using the add-in function of Palisade @RISK decision analysis software (Version 8.0), which operated directly on the preexisting mathematical nutrition models. The relative impact of changes in feed ingredient inclusion rates and water intake rates on RDS content and eNDF levels were evaluated for grower and finisher diets. The inclusion and intake rates for water and all feedstuffs were subjected to −25% to 25% variation in 75 steps. The analyses for RDS content excluded all non-S-containing feed ingredients, including VTM supplements and fat. Likewise, the analyses for eNDF levels excluded all non-fiber ingredients, such as LFB, fat, VTM supplement, and well water. Each analysis generated graphical and tabular results that ranked the critical factors from most to least, further highlighting the feedstuffs and/or district water wells that were directly responsible for excess S exposure and subsequent increased risk of S-PEM.

## 3. Results

### 3.1. Observed S Content

**Figure 1:**
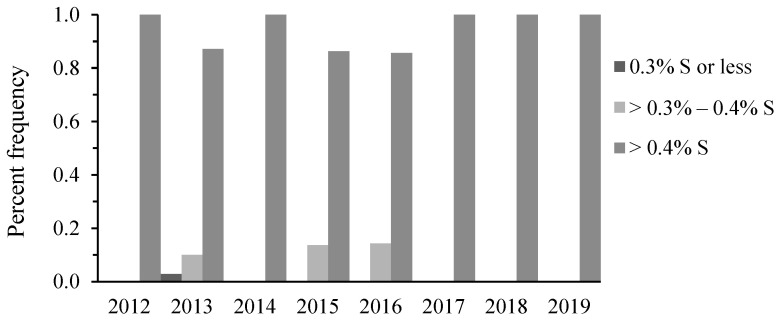
Percentage frequency of S content in Texas DDGS.

The S content in Texas DDGS samples ranged from 0.24% to 1.1%, with an average of 0.69% on a DM basis (Table 4). Based on OTSC data, the majority of these samples exceeded the NRC-recommended maximum tolerable levels (MTLs) of 0.3 to 0.4% S in beef (Figure 1). As shown in Table 1, the S content of all other feed ingredients was less variable than that in the DDGS, ranging from 0.09% to 0.26% DM.

**Figure 2:**
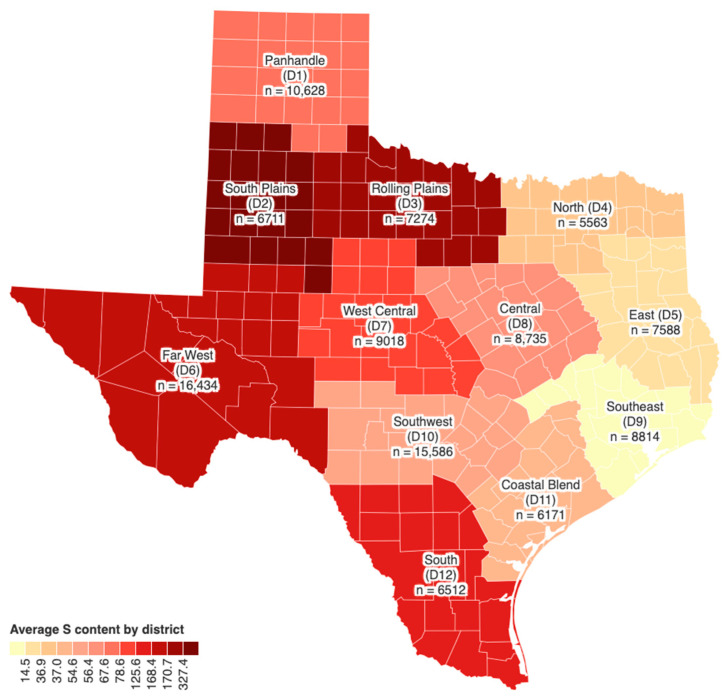
S content in Texas water wells categorized by districts outlined by Texas A&M AgriLife Research Extension Service.

Figure 2 displays the S levels in Texas water wells by district. The NRC recommends a MTL of 834 mg/L S in water for growing cattle and 200 mg S/L for finishing cattle. Based on this data, the average S content in well water across all districts was below the MTL for growers, while the S content in the South Plains district (D2) exceeded the MTL recommendation for finishers.

### 3.2. Risk Characterization

**Figure 3:**
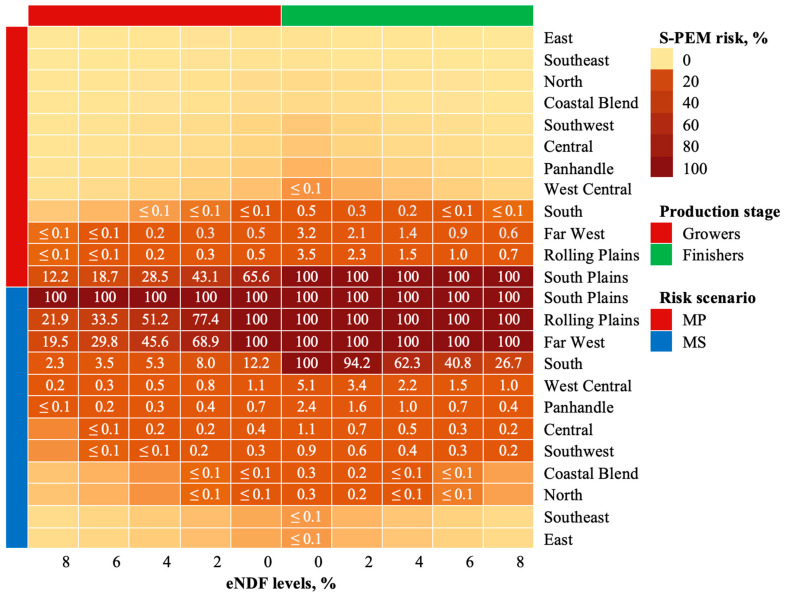
Heat map of S-PEM risk for Texas beef.

Figure 3 shows a heat map summarizing the most potential and significant S-PEM risk at five different eNDF levels for growers and finishers raised across twelve districts in Texas. Table 5 presents the RDS concentrations in DDGS-based diets, estimated utilizing conditions outlined in the MP and MS risk scenarios. Under MP risk conditions, RDS concentrations exceed the NRC-recommended MTL of 0.4% DM for growers in six of the twelve districts, while RDS content in ten of the twelve surpassed the 0.3% DM MTL for finishers. Under the MS risk scenario, RDS content for cattle in all districts exceeded the NRC-recommended MTLs, highlighting the potential health concern for Texas beef when DDGS inclusion rates reached 30%.

### 3.3. Sensitivity Analysis

**Figure 4:**
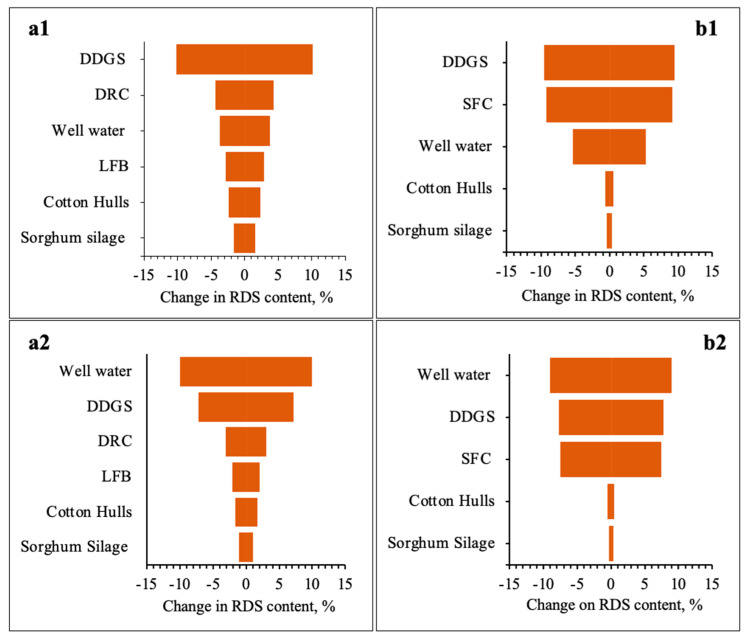
Sensitivity analysis for the low and high impact of well water on RDS content in formulated grower (**a1**,**a2**) and finisher (**b1**,**b2**) diets, respectively.

**Figure 5:**
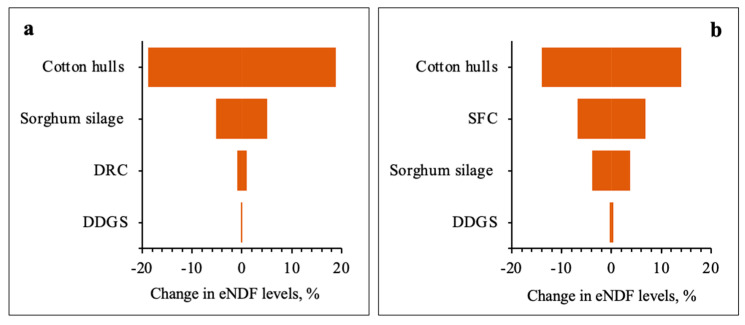
Sensitivity analysis of eNDF levels in formulated grower (**a**) and finisher (**b**) diets.

Figure 4 and Figure 5 show the relative impact on dietary RDS concentrations and eNDF levels caused by varying the feed ingredient inclusion rates and water intake rates by 25%. The sensitivity analysis of RDS content highlighted DDGS as the main contributor for growers and finishers in the East and Southeast districts, where the S content in water wells was ≤14.5 mg/L. Yet, for all other districts, well water was the most significant contributor of RDS content.

As expected, the sensitivity analysis of eNDF levels identified cotton hulls, the source of dietary roughage, as the major contributor of fiber for growers and finishers.

## 4. Discussion

Cattle production significantly contributes to Texas and the nation’s economy, providing employment opportunities and producing beef and beef-related consumable products that stimulate the entire market value chain and generate revenue. Nutrients and minerals found in feedstuffs and water play a vital role in many biological processes, helping ranchers ensure a desirable beef quality is achieved to maximize profit. Nutrient imbalances, such as excess S, have been historically shown to impact cattle health and subsequent economic performance in the central and western regions of the nation, like South Dakota, Nebraska, and Colorado [3,10,18]. Table 5 confirms that S concentrations in Texas DDGS were consistent with those reported by the NRC and other scientific peer-reviewed research performed throughout the nation. While S toxicity has been investigated in these areas, this work serves as the first attempt to assess the risk for Texas beef since the greater dietary inclusion of DDGS.

The NRC recommend MTLs of S in diets and water for beef animals, based on production stage and diet composition. Generally, cattle fed high-fiber diets have a higher tolerance for S, as shown in Table 6. The observed and analyzed S data reveal that the frequency of S in Texas DDGS and water wells typically exceeded 0.4% DM, the highest MTL for beef, emphasizing the significance of this research. More specifically, Figure 1 shows that S content in over 90% of Texas DDGS exceeded 0.4% DM over an eight-year consecutive period. In contrast, S content in only one (≤8%) of the twelve districts exceeded the 200 mg/L S MTL for finisher cattle. Figure 3 further indicates that feedlots located in West, Central, and South Texas encountered higher levels of S in water wells than those in the East. Historically, these areas also have the densest populations of concentrated animal feeding operations, where increased greenhouse gas emission has the potential to contaminate water resources [19].

Under the conditions of this research, finishers located in the South Plains district were found to be the most vulnerable beef population in Texas. In the MP scenario, growers in all districts except the South Plains encountered a range of 0.28% to 0.74% RDS, posing a ≤0.5% risk of S-PEM at all eNDF levels. For finishers, RDS concentrations reached 0.92%, causing a slightly higher S-PEM risk of ≤3.5%. Growers under conditions of the MP scenario were exposed to 1.19% RDS, resulting in a 12 to 66% risk of S-PEM, while finishers exposed to 1.56% RDS faced a 100% risk of S-PEM at all eNDF feeding levels. Observations of the MS risk scenario showed that increasing the DDGS inclusion rate to 30% and maximizing water intake rates substantially increased the S-PEM risk for all feeding groups. More specifically, RDS exposure for growers ranged from 0.51 to 1.19% DM, while finishers faced a potential exposure of 0.53 to 2.6% DM. However, expert opinion suggests that it is very unlikely for DDGS to reach 30% in feedlot rations. That in mind, coupled with the maximum level of S in all other feed ingredients, it is implied that ingredients used to prepare rations for Texas beef cattle present a minimal risk of S toxicosis.

Findings of the sensitivity analyses revealed that increased intake of water containing S exacerbated the S-PEM for all districts. For example, well water ranked second for its impact on RDS content for growers and finishers when S in water wells was ≤14.5 mg/L. Yet, when S concentrations in water wells surpassed 40 mg/L for growers and 30 mg/L for finishers, well water became the most significant contributor of RDS content, suggesting S content in Texas well water may be a more prominent concern than S in Texas DDGS. The sensitivity analysis of eNDF levels highlighted cotton hulls as the superior contributor of dietary fiber for growers and finishers, with major differences observed in the rankings of the energy feeds, like SFC and DRC. For instance, cotton hulls contributed the most significant eNDF levels for growers at roughly 19%, followed by sorghum silage at 5%, and DRC and DDGS at ≤1%. Yet, in finisher diets, cotton hulls at 14% were followed by SFC at 7%, sorghum silage at 4%, and DDGS at ≤1%. This difference is thought to have been heavily influenced by the high SFC inclusion rate of 68.2%. The scientific literature reports that SFC increases ruminal digestibility by 10%, the net energy of maintenance (NE_m_) by 15%, and the net energy of gain (NE_g_) by 19% [20,21]. Hence, the substitution of SFC for DRC and limited DDGS feeding for finishers appears to be a strategic approach for limiting DDGS and mitigating the potential risk of S-PEM.

Together, these findings support the existing theory that finishing cattle are most susceptible to S toxicosis due to the decreased consumption of forage fiber, preventing ruminal protection against H_2_S accumulation. Feedstuffs like SFC and DRC with low S content yet higher levels of eNDF have been emphasized as feed ingredients to balance high S sources, like DDGS. This research provides tools translatable nationwide that advance the understanding of dietary S exposure for beef, further helping mitigate the risk and maximize subsequent revenue from the production, manufacturing, and labeling of beef and feed ingredients.

## 5. Conclusions

Results revealed that the highest risk of S-PEM for Texas feedlot is posed to those in the South Plains district. Findings also highlight that when S content in well water exceeds 14.5 mg/L, water becomes the greatest contributor to dietary S content. Major outcomes of the entire S research demonstrated that it is possible to counterbalance excess S in beef diets by incorporating more fiber sources effective in stimulating ruminal activity or processed energy feeds that support ruminal digestion.

## Figures and Tables

**Table 1:** Nutrient composition of beef cattle feed ingredients.

Feed Ingredients ^1^	Sulfur, % DM	CP, % DM	RDP, % CP	Met, % RUP	NDF, % DM	eNDF, % NDF
Protein feeds						
DDGS	0.24 to 1.1	29.7	45.1	1.20	23.0	4.0
Energy feeds						
SFC	0.14	9.8	44.7	1.12	9.0	48.0
DRC	0.11 to 0.14	9.8	44.7	1.12	10.8	30.0
Roughage sources						
Sorghum silage	0.12	9.4	73.0	0.75	60.8	81.0
Cotton hulls	0.09	4.1	50.0	1.91	90.0	100.0
Supplements						
VTM supplement	-	-	-	-	-	-
LFB	0.26	-	-	-	-	-
Fat	-	-	-	-	-	-

CP = crude protein; RDP = rumen degradable protein; Met = methionine; NDF = neutral detergent fiber; eNDF = effective neutral detergent fiber. ^1^ DM = dry matter, DDGS = dried distillers grains with solubles, SFC = steamed flaked corn, DRC = dry rolled corn, VTM = vitamin–mineral supplement, LFB = liquid feed blend

**Table 2:** Formulas of mathematical nutrition models.

Product, % Diet DM	Equation
Rumen undegradable protein (RUP)	=100 − [(RDP, % CP) × (CP % DM)]
Amount of organic S in S-containing amino acids (SAA)	=(Ingredient inclusion rate, % diet DM) × (Met, % RUP) × (RUP, % DM) × (S in Met, % DM)
Total S in water wells (TS)	=(Average S in water well by district, mg/L) × (Water intake, L) × (1/10,000)
Total dietary S (TDS)	=[(Ingredient inclusion rate, % diet DM) × (Avg. Organic + Inorganic S)] + (TS in H_2_O wells)
Rumen-degradable S (RDS) ^1^	=TDS − (RUP × SAA)

^1^ RDS formula adopted from [11].

**Table 3:** Composition of DDGS-based diets used in MP and MS risk scenarios.

	MP Risk		MS Risk	
Item	Growers	Finishers	Growers	Finishers
Ingredient, % DM ^1^				
DDGS	18.1	16.8	30.0	30.0
SFC	-	68.2	-	55.0
DRC	36.1	-	24.3	-
Sorghum silage	13.5	3.3	13.5	3.3
Cotton hulls	27.1	6.7	27.1	6.7
VTM supplement	2.2	1.8	2.2	1.8
LFB	2.9	-	2.9	-
Fat	-	3.2	-	3.2
Water intake, L	29.0	40.8	47.0	66.0
Calculated nutrient content, % DM ^2^				
CP	11.5	12.3	13.9	14.9
NDF	40.7	18.0	42.1	19.9
eNDF	32.4	10.7	32.1	10.3

^1^ DM = dry matter, DDGS = dried distillers grains with solubles, SFC = steamed flaked corn, DRC = dry rolled corn, VTM = vitamin–mineral supplement, LFB = liquid feed blend. ^2^ CP = crude protein, NDF = neutral detergent fiber, eNDF = effective neutral detergent fiber.

**Table 4:** S content in DDGS reported by the NRC, published literature, and OTSC.

Item	Average S Content in DDGS ± SD, % DM	Range of S Content in DDGS, % DM	No. of Samples
NRC Tables			
NRC 2000 [4]	0.44 ± 0.12	-	113
NRC 2001 [14]	0.44 ± 0.15	-	278
Published Literature			
Batal and Dale, 2003 [15]	0.84 ± 0.21	0.45 to 1.10	9
Buckner et al., 2011 [16]	0.77	0.71 to 0.84	1200
Kim et al., 2012 [17]	0.65 ± 0.19	0.33 to 1.04	35
Laboratory			
OTSC	0.69 ± 0.19	0.24 to 1.1	331

**Table 5:** Estimated RDS content in Texas beef diets.

	MP Risk		MS Risk	
	RDS Content in Cattle Diets, % DM			
District	Growers	Finishers	Growers	Finishers
East	0.28	0.28	0.51	0.53
Southeast	0.28	0.28	0.51	0.53
North	0.35	0.37	0.62	0.68
Coastal Blend	0.35	0.37	0.62	0.68
Southwest	0.40	0.44	0.70	0.79
Central	0.40	0.45	0.71	0.81
Panhandle	0.44	0.50	0.76	0.88
West Central	0.47	0.54	0.81	0.95
South	0.60	0.73	1.03	1.26
Far West	0.73	0.91	1.23	1.55
Rolling Plains	0.74	0.92	1.25	1.56
South Plains	1.19	1.56	1.98	2.60

**Table 6:** MTLs of S in feedstuffs and water for beef cattle.

	Diet Composition			
Stage of Production	Forage Fiber	Concentrate	Dietary MTL, % DM	Water MTL, mg/L
Growing	Medium (30–80%)	Medium (20–70%)	0.4	834
Finishing	Low (5–30%)	High (70–95%)	0.3	200

MTL = maximum tolerable level; DM = dry matter.

## Data Availability

Data are contained within the article and Supplementary Materials.

## Supplementary Materials

The following supporting information can be downloaded at: https://www.mdpi.com/article/10.3390/ani14162400/s1, Table S1: S content in Texas DDGS reported by OTSC; Table S2: S content in Texas water wells categorized by 12 districts outlined by Texas A&M AgriLife Research Extension Service; Table S3: Probabilistic risk model for growers; Table S4: Probabilistic risk model for finishers.

## Abbreviations

BW, body weight; CP, crude protein; Cys, cysteine; D, district; DDGS, dried distiller grains with solubles; DM, dry matter; DRC, dry rolled corn; eNDF, effective neutral detergent fiber; HS-, bisulfide; H2S, hydrogen sulfide; ICP-OES, inductively coupled plasma–optical emission spectrometry; Met, methionine; MP, most potential; MS, most significant; MTL, maximum tolerable level; NDF, neutral detergent fiber; NEg, net energy of gain; NEm, net energy of maintenance; NRC, National Research Council; OTSC, Office of the Texas State Chemist; PEM, polioencephalomalacia; RDS, rumen-degradable sulfur; RDP, rumen-degradable protein; RUP, rumen-undegradable protein; S, sulfur; SAA, sulfur-containing amino acids; SCHO, structural carbohydrates; SFC, steam flaked corn; SO42-, sulfate; S-PEM, sulfur-induced polioencephalomalacia; TDS, total dietary sulfur; TS, total sulfur; VTM, vitamin–mineral supplement.
